# Computer-Aided Discovery of Small Molecules Targeting the RNA Splicing Activity of hnRNP A1 in Castration-Resistant Prostate Cancer

**DOI:** 10.3390/molecules24040763

**Published:** 2019-02-20

**Authors:** Lavinia A. Carabet, Eric Leblanc, Nada Lallous, Helene Morin, Fariba Ghaidi, Joseph Lee, Paul S. Rennie, Artem Cherkasov

**Affiliations:** Vancouver Prostate Centre, University of British Columbia, 2660 Oak Street, Vancouver, BC V6H 3Z6, Canada; lcarabet@prostatecentre.com (L.A.C.); eleblanc@prostatecentre.com (E.L.); nlallous@prostatecentre.com (N.L.); hmorin@prostatecentre.com (H.M.); fghaidi@prostatecentre.com (F.G.); jlee@prostatecentre.com (J.L.); prennie@prostatecentre.com (P.S.R.)

**Keywords:** hnRNP A1, alternative splicing, castration-resistant prostate cancer, computer-aided drug discovery, small molecule inhibitors, protein–RNA interactions

## Abstract

The heterogeneous nuclear ribonucleoprotein A1 (hnRNP A1) is a versatile RNA-binding protein playing a critical role in alternative pre-mRNA splicing regulation in cancer. Emerging data have implicated hnRNP A1 as a central player in a splicing regulatory circuit involving its direct transcriptional control by c-Myc oncoprotein and the production of the constitutively active ligand-independent alternative splice variant of androgen receptor, AR-V7, which promotes castration-resistant prostate cancer (CRPC). As there is an urgent need for effective CRPC drugs, targeting hnRNP A1 could, therefore, serve a dual purpose of preventing AR-V7 generation as well as reducing c-Myc transcriptional output. Herein, we report compound VPC-80051 as the first small molecule inhibitor of hnRNP A1 splicing activity discovered to date by using a computer-aided drug discovery approach. The inhibitor was developed to target the RNA-binding domain (RBD) of hnRNP A1. Further experimental evaluation demonstrated that VPC-80051 interacts directly with hnRNP A1 RBD and reduces AR-V7 messenger levels in 22Rv1 CRPC cell line. This study lays the groundwork for future structure-based development of more potent and selective small molecule inhibitors of hnRNP A1–RNA interactions aimed at altering the production of cancer-specific alternative splice isoforms.

## 1. Introduction

hnRNP A1 is a multifunctional RNA-binding protein that regulates alternative pre-mRNA splicing, transcription, nucleocytoplasmic shuttling, miRNA processing, and telomere elongation maintenance, as well as translation of cellular transcripts both in physiological and pathological conditions [1,2]. Overexpression of hnRNP A1 in various cancer types, including prostate [3,4], lung [5], stomach [6], and breast [7] cancers, Burkitt lymphoma [8], multiple myeloma [9], leukemia [10] and neuroblastoma [11], has been associated with tumorigenesis, cancer progression and drug resistance. The molecular mechanisms by which hnRNP A1 supports malignant transformation, either directly or through interplay with well-established cancer drivers, include regulation of cell survival, alteration of cell cycle, invasion and metastasis, altered metabolism and stress adaption (i.e., to hypoxia, starvation, and response to DNA damage), all of which are recognized hallmarks of cancer [1,2,12].

In prostate cancer (PCa), the second leading cause of cancer-related death in men, alternative splicing (AS) plays a prominent role as it represents a mechanism of resistance to therapy [3]. There is accumulating evidence on hnRNP A1 splicing regulatory interconnectivity with the androgen receptor (AR) and c-Myc oncogenic signaling pathways. On one hand, AR, a ligand-activated transcription factor is a driving force in PCa and constitutes the main drug target for advanced cases of disease [13]. Despite the initial efficacy of androgen deprivation therapy, tumors recur resulting in castration-resistant prostate cancer (CRPC) which often remains dependent on AR activity [14]. A well-described mechanism of resistance to treatment with second generation AR-directed therapies is aberrant splicing of AR pre-mRNA that yields highly active AR splice variants (AR-V) which lack the AR ligand-binding domain and thus are unresponsive to the antiandrogen treatments [15]. The constitutively active, ligand-independent AR-V7 splice isoform, for instance, has been shown to confer primary resistance to enzalutamide—a potent AR antagonist, and to abiraterone—an inhibitor of androgen synthesis [16,17]. AR-V7 knockdown with antisense nucleotides has been shown to suppress the growth of the enzalutamide-resistant 22Rv1 CRPC cell line [18]. On the other hand, in CRPC, AR is co-expressed with c-Myc oncogenic transcription factor, which can induce androgen-independent growth and activate AR in a ligand-independent manner [19]. The well-characterized overexpression of c-Myc in cancer [20] also affects critical splicing programs. Notably, it has been demonstrated that under direct transcriptional control of c-Myc, in CRPC, hnRNP A1 promotes the production of the AR-V7 splice variant [4,12,21,22], which in turn directly regulates the expression level of key proteins involved in cell-cycle progression and proliferation, in particular the ubiquitin conjugating enzyme 2C (UBE2C) in androgen-deprived 22Rv1 cells [23,24]. In CRPC, knockdown of hnRNP A1 with short-interfering RNA (siRNA) has been shown to suppress AR-V7 levels and growth of 22Rv1 cells [4].

Furthermore, in cancer, hnRNP A1 contributes to aerobic glycolysis by promoting the expression of the pyruvate kinase PKM2 isoform, a hallmark of tumor growth, as well as ∆Max, a Myc-associated factor X (Max) isoform, enhancer of Myc-dependent transformation and mediator of Myc-dependent tumor metabolism [25,26]. Moreover, it is also notable that while hnRNP A1 is transcriptionally upregulated by c-Myc, it is also one of the factors known to act on the c-Myc promoter to regulate c-Myc transcription [27,28] and, moreover, to directly promote translation of c-Myc protein itself by binding to internal ribosome entry sites (IRES) found in the 5′-UTR (untranslated region) of c-Myc mRNA [29].

How hnRNP A1 discerns among its various functions thus depends on recognition and binding to specific RNA sequences (also DNA for functions such as transcription or telomere elongation). While hnRNP A1 was characterized early as a splicing repressor binding to exonic or intronic splicing silencers, recent evidence indicates that hnRNP A1 more globally affects alternative splicing outcomes by binding directly to 3′-splice sites (3′ss) that contain consensus 5′-YAG-3′ motifs [30,31,32]. It has been demonstrated that the AR-V7 splice variant is generated via splicing at the alternative 3′ss next to the cryptic exon 3B which includes a stop codon, rather than the 3′ss next to AR exon 4, resulting in the translation of the AR-V7 C-terminal-truncated form of AR [21,33]. Importantly, emerging data suggest that the recruitment of hnRNP A1 to the AR-V7 3′ss in AR pre-mRNA is increased in 22Rv1 cells with acquired enzalutamide resistance [4], and that the specificity of hnRNP A1–RNA binding, as determined by X-ray crystallography, is dictated by the 5′-AG-3′ dinucleotide [34].

Structurally, hnRNP A1 has a modular domain organization consisting of tandem N-terminal RNA recognition motifs (RRM1 and RRM2, respectively), collectively referred to as unwinding protein 1 (UP1), and an intrinsically disordered Glycine-rich C-terminal region. The C-terminus mediates homologous and heterogeneous protein–protein interactions. The Gly rich C-terminal region contains an Arg-Gly-Gly (RGG) box which is involved in non-specific RNA binding, and a nuclear targeting sequence, termed M9, which is responsible for bidirectional nucleocytoplasmic shuttling. UP1 is the primary RNA-binding domain (RBD) involved in specific protein–RNA interactions and alternative splicing events [1,30,34].

To date, inhibition of hnRNP A1 has been demonstrated with the naturally occurring flavonoid quercetin, a rather promiscuous, poorly soluble and poorly bioavailable compound [35,36,37]. Identified as an hnRNP A1 binder through chemical proteomics, quercetin exerted nonetheless anti-cancer effects in PC3 AR-independent PCa cell line by a mechanism of action that involves binding to the C-terminal region of hnRNP A1, impairment of hnRNP A1 ability to shuttle between the nucleus and cytoplasm, resulting in its cytoplasmic retention and accumulation, driving cells toward apoptosis [38]. More recently and pertinent to this work, quercetin has been shown to downregulate hnRNP A1 and thus AR-V7 expression in CRPC cell lines, and to re-sensitize enzalutamide-resistant 22Rv1-injected mouse xenografts in vivo [39].

## 2. Results

In this study, we report compound VPC-80051, a novel hnRNP A1 small molecule inhibitor targeting the hnRNP A1 RBD, the first to be identified using a computer-aided drug discovery approach.

### 2.1. Binding Site Identification on hnRNP A1 RBD

The published 1.92 Å X-ray structure of the UP1 RNA-binding domain (RBD) of hnRNP A1 bound to its RNA 5′-AGU-3′ trinucleotide target sequence (PDB ID: 4YOE) [34] was utilized for identification of plausible pockets where small molecule inhibitors could bind and specifically disrupt hnRNP A1 binding to RNA and, therefore, to interfere with the subsequent splicing activities. The 4YOE X-ray structure provided the first insights into the specificity of hnRNP A1–RNA recognition which indicated that UP1 specifically interacts with the 5′-AG-3′ ribodinucleotide within a nucleobase cavity formed upon folding of the RRM1 motif and the inter-RRM linker. RRM2 made no contact with the RNA sequence. Both A (adenine) and G (guanine) purines engaged in stereospecific contacts within the pocket thus explaining the preference for the 5′-AG-3′ sequence [34]. Their replacement for pyrimidines, having smaller ring size, were deemed unlikely to satisfy the favorable interactions of the purines with similar energetics. For instance, single or double substitutions for cytosine resulted in a ~20-fold reduction in UP1 RNA-binding affinity, rate-limiting for complex formation [30,34,40].

**Figure 1:**
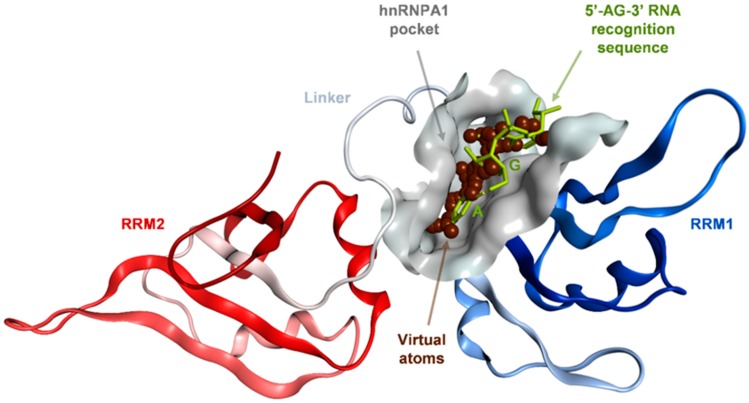
In silico model of the UP1 domain of the hnRNP A1 splicing factor bound to the 5′-AG-3′ recognition sequence constructed based on the 1.92 Å X-ray structure of RNA-bound UP1 (PDB ID: 4YOE). The RNA bases are shown as sticks and colored in green. The predicted binding site on hnRNP A1 at the RNA recognition interface is represented as a grey solid surface. Virtual atoms utilized to probe the protein surface are shown as brown spheres within the identified pocket. RRM: RNA recognition motif.

Based on the prior crystallographic evidence regarding the structural determinants of hnRNP A1–RNA recognition specificity, we selected the RRM1 and the inter-RRM linker regions of UP1 for binding site identification. We employed the Site Finder module of the Molecular Operating Environment (MOE) suite of programs [41], which predicted a binding site (Figure 1) significantly matching the X-ray defined nucleobase pocket. 

**Figure 2:**
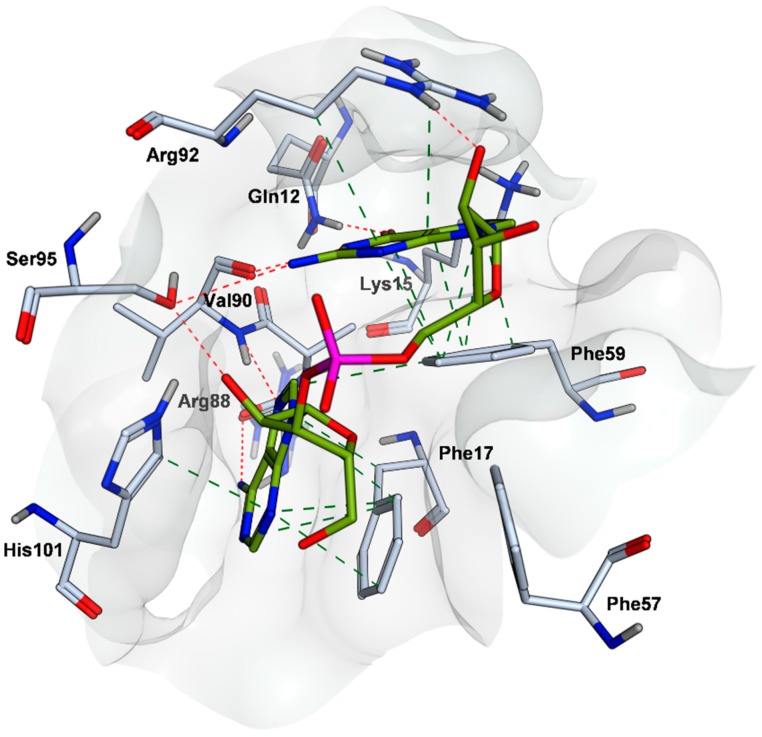
hnRNP A1 interactions with 5′-AG-3′ RNA recognition sequence within the pocket. Hydrogen-bonding interactions formed between the nucleobases with the backbone and side chains of Val90, Arg88, Gln12 residues, as well as those formed by the sugars of the RNA backbone with side chains of Arg92 and Ser95 are indicated with red dashed lines. Hydrophobic interactions with Phe17, His101, Phe59 and aliphatic side chains are indicated with dashed green lines. Inhibitors targeting the pocket are expected to block UP1 binding to RNA and alter hnRNP A1 splicing activity.

The predicted binding site is shaped by residues: Gln12, Lys15, Phe17, Met46, Arg55, Phe57, Phe59, Lys87, Arg88 from the RRM1 motif, and Ala89, Val90, Ser91, Arg92, Ser95 and His101 from the inter-RRM linker. The functional relevance of these residues has been well-described (Figure 2) [34]. Phe17 and His101 contribute to the majority of binding free energy, originating from favorable van der Waals and π-π stacking interactions with the rings of the adenine at first position in the RNA 5‘-AG-3‘ sequence. Moreover, Val90 and Arg88 backbone amides make specific H-bonding interactions with the adenine nitrogen atoms. Additional H-bonding and hydrophobic packing are contributed from the hydroxyl group of Ser95 and Phe57, respectively. At the second 5′-AG-3′ RNA position, amino groups of Gln12 and Lys15 primarily and selectively recruit the guanine through H-bonding via their amino group. In addition, Val90 and Arg92 provide H-bonding capabilities via their backbone carbonyl and guanidinium groups. Cation-π and π-π interactions with Arg92 and Phe59, respectively, further enhance the interactions with the guanine [34].

Small molecule inhibitors targeting this site are expected to interfere with the most functionally relevant protein–RNA interactions within the site to alter hnRNP A1′s splicing activity.

### 2.2. In Silico Identification of Hit Compounds Targeting the hnRNP A1 RBD Site

Subsets of drug-like molecules deposited in the ZINC15 open chemical repository [42,43,44] having in-stock availability from selected vendor catalogues, filtered by logP (octanol-water partition coefficient), charge and chemical reactivity, were virtually screened against the identified hnRNP A1–RNA pocket utilizing Glide docking software [45,46] in standard precision (SP) mode as a primary structure-based screening technique. The compounds with the best docking scores were prioritized for subsequent in silico scoring based on calculated pK_i_ [47] and RMSD (root mean square deviation) between poses obtained from various docking programs, including ICM [48] and Hybrid [49]. As a result, 139 chemicals were selected for purchase based on two or more of the abovementioned binding affinity indicators and satisfaction of at least three essential π-π or H-bonding interactions with the residues shaping the pocket, in particular contacts with Phe17, His101, Val90 and Phe59.

These compounds were then subjected to experimental evaluation through the UBE2C reporter assay (see Section 4.5) for assessing their effect on AR-V7 driven luciferase-detected UBE2C activity in 22Rv1 cells in androgen-deprived conditions, with quercetin, the previously reported hnRNP A1 binder used as positive control. A dozen compounds demonstrated more than 50% inhibition of UBE2C activity at a 25 μM concentration in this assay.

### 2.3. In Vitro Characterization of Hit Compound VPC-80051

**Figure 3:**
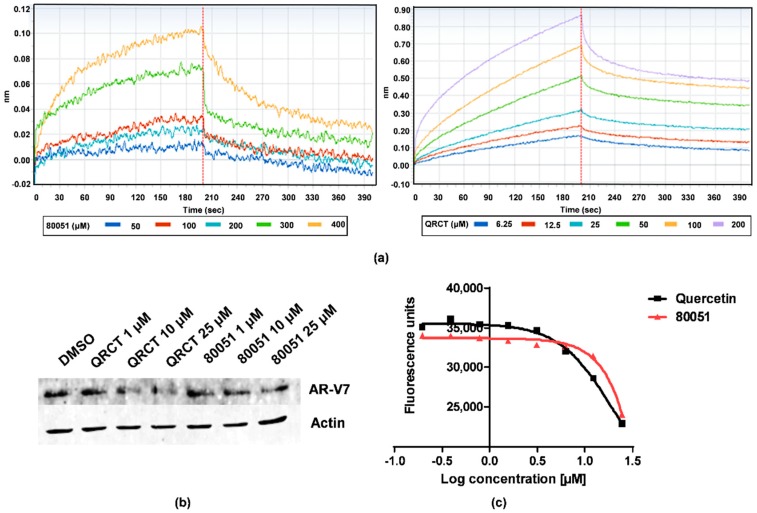
In vitro characterization of VPC-80051 hnRNP A1 inhibitor. (**a**) Dose-dependent direct binding of VPC-80051 and control quercetin (QRCT) to purified UP1 domain of hnRNP A1 protein quantified by bio-layer interferometry (BLI). (**b**) Reduction of levels of AR-V7 splice variant upon treatment with 10 and 25 μM of VPC-80051 and QRCT as analyzed by Western blotting. (**c**) Reduction of 22Rv1 cell viability upon treatment with VPC-80051 and quercetin at greater than 10 μM doses where decrease in AR-V7 levels was observed.

To quantify the ability of hit compounds to interact directly with hnRNP A1 RBD, the Bio-Layer Interferometry (BLI) technique was employed with purified UP1 domain of the hnRNP A1 protein (N-terminal residues 1-196) immobilized on a streptavidin biosensor (see Section 4.6 for details). Compound VPC-80051, as well as quercetin control, demonstrated direct binding to hnRNP A1 in a dose-dependent manner (Figure 3a).

Quantitative RT-PCR (qRT-PCR) was further employed to assess the effect of hits on the mRNA levels of AR-V7 in 22Rv1 cells in androgen-deprived conditions. Treatment with VPC-80051 at 25 and 10 μM concentrations resulted in a reduction of AR-V7 levels comparable to that of quercetin. The ratios of measured cycle threshold differences (∆CT) of levels of AR-V7 mRNA versus DMSO control normalized to actin, in percentage, were 66.20% and 79.55% for VPC-80051 at 25 and 10 μM, respectively, compared to 62.25% and 71.15% for quercetin at same concentrations. The reduction of AR-V7 levels observed in qRT-PCR upon 24 h treatment with VPC-80051 and quercetin was corroborated by Western blot analysis (Figure 3b). Furthermore, the decrease in AR-V7 levels correlated with a reduction in viability of 22Rv1 cells treated with VPC-80051 at tested concentrations (Figure 3c).

### 2.4. Mode of Binding of VPC-80051 to hnRNP A1 RBD Site

**Figure 4:**
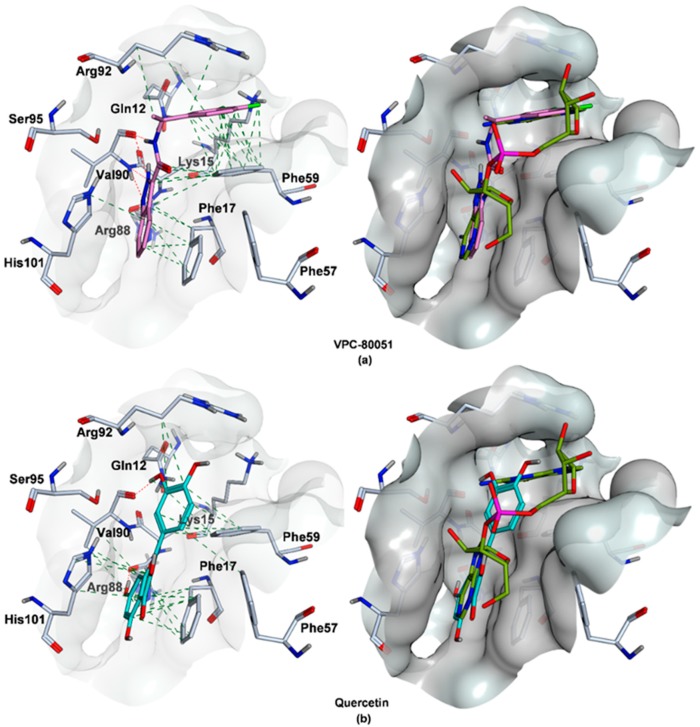
(**a**) Left. Predicted binding pose of VPC-80051 within the hnRNP A1 RBD pocket. VPC-80051 is shown in stick representation (magenta = carbon, blue = nitrogen, red = oxygen, green = fluorine). Interacting residues are shown as sticks and colored in light blue-grey. The pocket surface is colored in light grey. Hydrogen bonds formed by VPC-80051 with the backbone amide and carbonyl of Val90 are indicated with red dashed lines. Hydrophobic interactions with aromatic rings of Phe17, His101 and Phe59 as well as with aliphatic side-chains of residues shaping the pocket are indicated with green dashed lines; Right. Significant structural overlap between VPC-80051 and nucleobases of the 5′-AG-3′ RNA within the hnRNP A1 RBD pocket; (**b**) Left. Predicted binding pose of quercetin. Hydrogen bonds formed with Val90 and Arg88 backbone carbonyls are indicated with red dashed lines; Right. Structural overlap between quercetin and the 5′-AG-3′ RNA. Minimal overlap with the guanine base. VPC-80051 chemical name: (*N*-[(1S)-1-(3,4-difluorophenyl)ethyl]-1*H*-indazole-3-carboxamide). Quercetin chemical name: 2-(3,4-dihydroxyphenyl)-3,5,7-trihydroxy-4*H*-chromen-4-one.

The predicted binding pose of VPC-80051 is shown in Figure 4a. The chemical structure VPC-80051 is composed of an indazole bicyclic aromatic moiety at one end, a carboxamide linker and a difluorophenyl ring at the other end. Within the docking site at the hnRNP A1–RNA binding interface, the benzene ring of the indazole group of VPC-80051 forms π-π stacking interactions with both Phe17 and His101. Moreover, the pyrazole ring of the indazole group forms three hydrogen bonds with the backbone of Val90: two between the Val90 backbone amide with the pyrazole nitrogen atoms and a third with Val90 backbone carbonyl. In addition, a fourth hydrogen bond is formed between the amine hydrogen of the carboxamide linker and the backbone carbonyl of Val90. All these interactions made significant contributions to the binding specificity of hnRNP A1 to the adenine in the first position of the cognate 5′-AG-3′ RNA sequence, as described in Section 2.1. In the predicted docking pose, the indazole moiety of VPC-80051 was positioned such that it significantly overlapped that of the 5′ adenine in the X-ray structure with a calculated RMSD of 2.85 Å, taking over these specific interactions within the cavity (Figure 4a right). Furthermore, the difluorophenyl ring of VPC-80051 makes significant hydrophobic interactions with the aromatic ring of Phe59, as well as with the aliphatic side-chains of Arg92 and Lys15, overlapping to a good extent with the guanine at the second position in the 5′-AG-3′ recognition sequence with RMSD of 2.82 Å (Figure 4a right). The Glide score of VPC-80051 was −8.2 kcal/mol and its binding affinity was dominated by hydrophobic interactions as per calculated pK_i_ (4.5 out of 6.8). For comparison, the Glide score of the literature compound quercetin was −6.2 kcal/mol and its predicted pK_i_ was 6. Quercetin’s predicted binding pose is shown in Figure 4b. Quercetin forms two hydrogen bonds: one between a hydroxyl group attached to the chromen-4-one moiety at first position with the backbone carbonyl of Arg88 and a second between a hydroxyl of its catechol moiety at second position with the backbone carbonyl of Val90. Additionally, π-π interactions are formed between the chromen-4-one moiety of quercetin with Phe17 and His101. While the overlap between the trihydroxy-chromen-4-one group with the 5′ adenine of RNA is significant with an RMSD of 2.7 Å (Figure 4b right), there is little overlap at the second position between the catechol (dihydroxyphenyl) ring and the guanine base (RMSD = 4.3 Å).

Further confidence in the binding pose of VPC-80051 was given by calculated RMSD values of 1.4 and 1.1 Å between the Glide pose and the poses obtained from ICM and Hybrid docking programs, respectively. Of note, the RMSD with the best pose generated by a “blind docking” Glide SP calculation, in which conformational sampling occurs within a grid large enough to cover the entire UP1 domain of the hnRNP A1 protein and not only the smaller grid centered on selected RRM1 and inter-RRM residues of the predicted binding site, was 1.7 Å. This provided good evidence on the accuracy of the binding site model as well as on the affinity of VPC-80051 for the site having a large number of equivalent interactions to those formed by 5′-AG-3′ cognate RNA with the X-ray nucleobase pocket.

**Figure 5:**
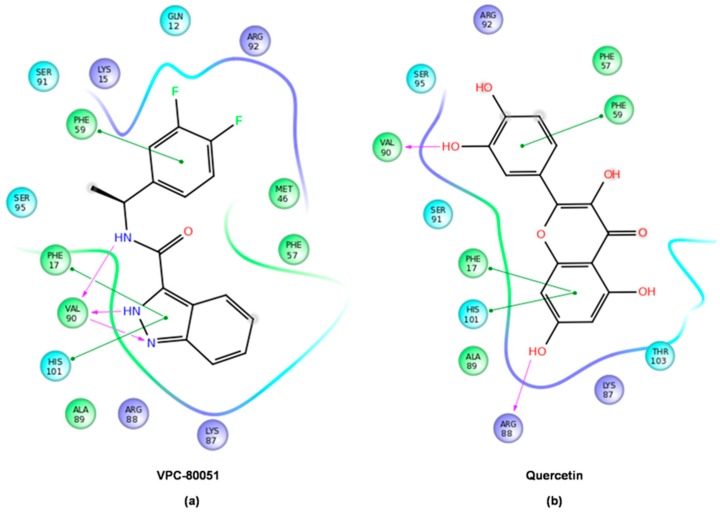
hnRNP A1-ligand interactions obtained from MM/GBSA simulations with implicit solvent for: (**a**) VPC-80051; and (**b**) quercetin in two-dimensional representation. Green lines indicate π-π interactions, purple arrow pointed lines represent backbone hydrogen-bonding interactions, and the curved contour represents the shape of the pocket around the ligand colored by the character of the interacting protein residues: (green = hydrophobic, blue = positively charged, cyan = polar).

The docked structures were further minimized in implicit solvent and the binding free energies were calculated with the MM/GBSA method (molecular mechanics with generalized Born and surface area continuum solvent model) [50]. The estimated binding free energy of VPC-80051 obtained from MM/GBSA simulations was −60.7 kcal/mol relative to −57.9 kcal/mol for quercetin control. In these simulations in which the binding site residues were allowed to relax (i.e., protein flexibility within 5.0 Å from the ligand) during the minimization procedure of the complexes, VPC-80051 and quercetin maintained the protein-ligand interactions predicted by rigid docking (Figure 5).

**Figure 6:**
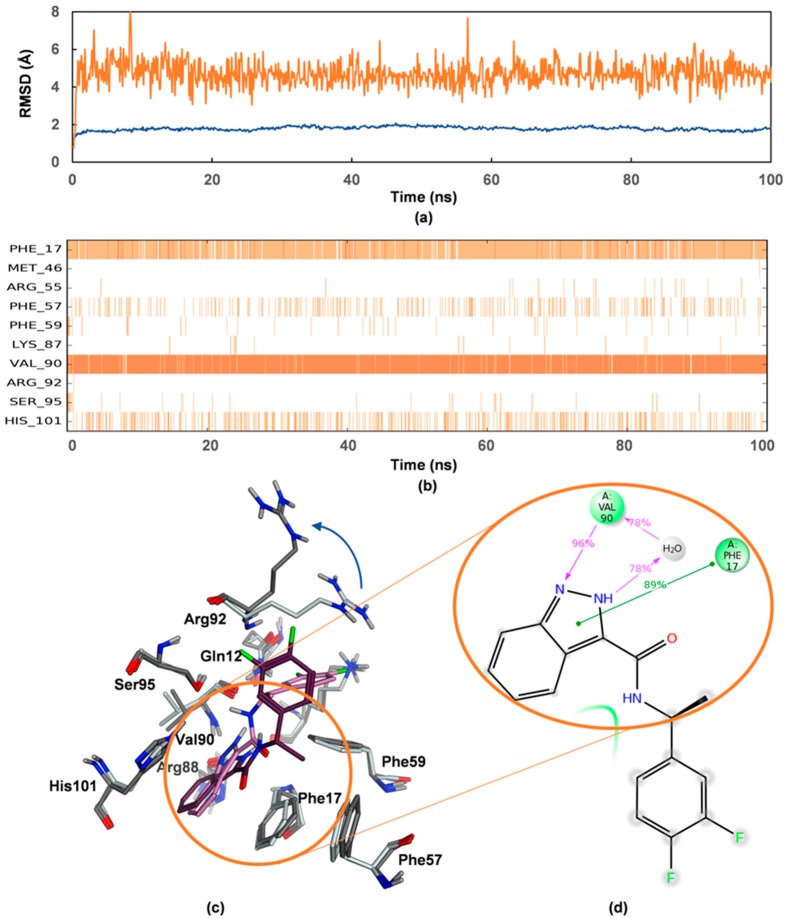
Dynamics of hnRNP A1-80051 complex during 100 ns MD simulations with explicit solvent. (**a**) RMSD displacement during the simulation trajectory of the protein side chains (blue) and of the ligand with respect to the protein (orange); (**b**) Protein-ligand contacts during the simulation time course. Val90 and Phe17 are main stabilizing residues with His101 and Phe57 seconding; (**c**) Three-dimensional superimposition of hnRNP A1 binding site and VPC-80051 at the beginning of the simulation and after 88.4 ns simulation time. Light grey and light magenta indicate the protein residues and the ligand in the reference frame while dark grey and dark magenta indicate the residues and the ligand in the selected 88.4 ns snapshot. The blue curved arrow indicates the larger movement of Arg92 side chain during the simulation; (**d**) Two-dimensional schematic of detailed interactions between VPC-80051 atoms with the protein residues. Stabilization of the ligand occurs via hydrogen bonding and water bridges with Val90 (purple arrow pointed lines) and π-π interactions with Phe17 (green lines). The percentages indicate the strength of ligand-protein interactions.

Computationally expensive molecular dynamics (MD) simulations were subsequently carried out to gain further insights into induced fit motions and solvent effects, and thus, the dynamic behavior of the hnRNP A1/VPC-80051 complex when fully subjected to force field in explicit solvent as opposed to the MM/GBSA implicit solvent approach. Analysis of the trajectory obtained from MD simulations (see Section 4.4) showed that VPC-80051 was stabilized for more than 30% of the 100 ns simulation time at greater than 80% strength mainly by hydrogen-bonding between its indazole moiety with Val90 backbone and π-π interactions with Phe17 (Figure 6). Superimposition of conformations of the simulated complex taken at different time steps revealed that the side chain of Arg92 underwent larger fluctuations relative to other side chains in the pocket (Figure 6c), opening and making the binding site more exposed to the solvent. As such, with more translational and rotational freedom, the difluorophenyl ring of VPC-80051 is no longer engaged in hydrophobic interactions with Arg92 side chain or π-π interactions with Phe59, and as such does not contribute substantially to the stabilization of the ligand. Future NMR spectroscopy or X-ray crystallography structural studies may unequivocally prove the binding mode of VPC-80051 to the hnRNP A1 RBD pocket.

## 3. Discussion

Here we presented VPC-80051, a small molecule prototype inhibitor of the hnRNP A1 splicing factor. To our knowledge, VPC-80051 is the first drug-like, non-promiscuous hnRNP A1 inhibitor discovered to date by computational approaches involving large-scale virtual screening targeting hnRNP A1 binding to RNA.

Unlike the literature-reported hnRNP A1 inhibitor quercetin, a well-known aggregator [51] and containing the catechol_A (92) moiety, one of the worst offenders enlisted in PAINS (Pan-Assay Interference Compounds) [52], annotated in more than 46 catalogs for promiscuous binding and various inhibitory activities against multiple targets [53] and as such rejected by FAF-Drugs4 ADME-Tox filtering tool [54] for inclusion in any drug discovery campaign, VPC-80051 contains no PAINS moieties and has no documented experimental activities against any protein target. Moreover, the quantitative estimate of druglikeness (QED) score [55] of VPC-80051 as calculated by FAF-QED web service [56] is 0.78 compared to 0.5 of quercetin. On a scale from 0 to 1, only compounds with a QED score above 0.65 (i.e., the median score for the current orally bioavailable drugs) are considered as having a desirable drug likeliness profile [55].

On the binding mode of VPC-80051 to the hnRNP A1 identified pocket, good agreement was obtained between various in silico techniques, including rigid docking with consensus scoring and flexible MD simulations with both implicit and explicit solvent. Furthermore, VPC-80051 showed evidenced ability to bind directly to the UP1 RNA-binding domain of hnRNP A1 in vitro and to alter hnRNP A1 alternative splicing activity in castrate-resistant 22Rv1 cells by reducing expression of the AR-V7 splice variant that promotes drug-resistance in CRPC.

By all means exploratory, this study provides a foundation for future computational studies that, when combined with rigorous biological validation, may lead to the discovery of novel, more potent and selective hnRNP A1 inhibitors. Combination studies with current anti-AR drugs or c-Myc inhibitors may provide synergistic or additive responses. Targeting the c-Myc/hnRNP A1/AR-V7 axis may be a viable strategy for treatment of CRPC. Targeting alternative splicing may offer future opportunities for development of next generation cancer-specific therapeutics.

## 4. Materials and Methods

### 4.1. Binding Site Identification on hnRNP A1 RBD

The published 1.92 Å X-ray structure of the UP1 RNA-binding domain of the hnRNP A1 alternative splicing factor bound to its RNA 5′-AGU-3′ trinucleotide target sequence (PDB ID: 4YOE) was subjected to the Site Finder module of the Molecular Operating Environment (MOE)—a fully integrated drug discovery software platform [41]. The Site Finder algorithm calculates plausible sites by scanning the protein surface with virtual atoms of either hydrophobic or hydrophilic character, which are subsequently clustered, scored and ranked based on size and propensity for ligand binding, an index accounting for amino acid composition at the protein-ligand interaction interface. One site was identified at the surface formed by the RRM1 and the inter-RRM linker regions of the UP1 RNA-binding domain of hnRNP A1 that matched to a large extent the X-ray described nucleobase pocket for the cognate 5′-AG-3′ RNA ligand [34].

### 4.2. Virtual Screening

Structure-based molecular docking was performed using the Glide program [45,46] (Maestro version 10.3.015, Schrӧdinger LLC, New York, NY, USA) [57] in SP mode, to screen ~4.3 million drug-like chemicals from ZINC15 repository [42,43,44]. Prior to docking, each chemical was washed and energy-minimized under the MMFF94x force field and Born solvation as per the ligand preparation protocol implemented in MOE [41]. In preparation for rigid docking, hnRNP A1 X-ray protein structure (PDB ID: 4YOE) [34] was prepared following Maestro’s standard protein preparation protocol. A docking grid was defined as a 30 Å box centered on the residues of the predicted hnRNP A1 RNA binding site for sampling and scoring of compounds. Molecules with Glide docking scores ≤ −6 kcal/mol were subjected to pK_i_ calculations using scoring.svl analysis tool for non-bonded intermolecular interactions [47]. Best scoring compounds were re-docked utilizing ICM [48] and OpenEye Hybrid [49,58] docking programs. The root mean square deviation (RMSD) in atomic coordinates between docking results obtained from various programs was calculated using the mol_rmsd.svl script [59]. RMSD was also calculated for poses obtained using the “blind docking” virtual screening technique. “Blind docking” was performed using Glide in SP mode on a large grid defined as a 76 Å box encompassing the entire UP1 domain of hnRNP A1 protein. 139 compounds were purchased based on favorable Glide scores, RMSD or pK_i_ indicators as well as satisfaction of important interactions with the pocket residues.

### 4.3. MM/GBSA Simulations

To obtain estimates of the binding free energies of compounds to the hnRNP A1 site, the docking poses were subjected to MM/GBSA simulations with implicit solvent as implemented in the Prime program of the Schrӧdinger software suite [60]. Each protein-ligand complex was MM minimized utilizing the OPLS3 force field [61] and the variable dielectric VSGB 2.0 solvent model [62]. During the minimization of the complexes, hnRNP A1 binding site residues within 5.0 Å of the ligand were allowed to undergo fluctuations to take into account the protein flexibility, while the rest of the protein structure was kept fixed.

### 4.4. Molecular Dynamics Simulations

Molecular dynamics (MD) simulations with explicit solvent of hnRNP A1-80051 complex were carried out utilizing Maestro’s Desmond MD package [63]. The MM/GBSA minimized complex was utilized as the initial structure. The system was neutralized by addition of 2 chlorine counterions and solvated utilizing the TIP3P water model. The system consisting of a total of 32491 atoms with 9856 water molecules was subjected to the OPLS3 force field under periodic boundary conditions. The system was allowed to relax prior to the 100 nanoseconds MD production run. MD simulations were conducted under the isothermal-isobaric ensemble with the temperature kept constant at 300 K using the Nosé-Hoover thermostat [64] and the pressure kept constant at 1 atm using the Martyna-Tobias-Klein barostat [65] with a relaxation time of 2 ps and isotropic coupling. The integrator used to evolve the system was RESPA (reference system propagator algorithm) [66] with a timestep scheduling of 2 fs for bonded and non-bonded van der Waals and short-range electrostatic interactions, and 6 fs for non-bonded long-range electrostatic interactions. The cutoff for the Coulombic short-range interactions was set to 9 Å. A harmonic restraint with a 1 kcal/mol force constant was imposed on the alpha carbons of the protein backbone. MD simulations were executed on the GPU-enabled Helios cluster of Compute Canada high-performance computing platform.

### 4.5. AR-V7 Level Measurement

As the AR-V7 splice variant has been shown to specifically regulate the expression levels of UBE2C in androgen-deprived 22Rv1 cells [24], a UBE2C reporter screening assay was developed in house to monitor the levels of the AR-V7 isoform in 22Rv1 cells by using a plasmid containing the UBE2C promoter linked to a luciferase reporter [67]. The UBE2C reporter plasmid was purchased from GeneCopoeia (product ID #HPRM16429). The Biolux Gaussia luciferase assay kit was purchased from New 20 England Biolab (#E3300L). 22Rv1 cells were plated with 10,000 cells per well in 96-well plates in RPMI media supplemented with 5% charcoal-stripped serum (CSS) and treated for 1 day with 1 μM, 10 μM and 25 μM of compounds.

### 4.6. Bio-Layer Interferometry Assay

The direct interaction between purified UP1 RNA-binding domain of the hnRNP A1 protein (N-terminal residues 1-196) and compounds was quantified with bio-layer interferometry (BLI) technique using OctetRED (ForteBio). hnRNP A1 was biotinylated in situ using an AviTag^TM^ sequence (GLNDIFEAQKIEWHE) (Avidity, LLC, Aurora, CO, USA) incorporated at the N-terminus of the hnRNP A1. *Escherichia coli* BL21 containing both biotin ligase and hnRNP A1 vectors were induced with 0.5 mM isopropyl-β-d-thiogalactopyranoside (IPTG) and the protein was expressed for 4 h at 25 °C in the presence of 125 μM biotin. The bacteria were then lysed by sonication, and the resulting lysate was purified by immobilized metal ion affinity chromatography (IMAC) with nickel–nitrilotriacetic acid (Ni–NTA) resin followed by size exclusion chromatography (superdex s75, GE Healthcare). The purified hnRNP A1 at 0.1 mg/mL was immobilized on the streptavidin sensors (SA) overnight at 4 °C. The sensors were then blocked, washed and moved into wells containing various concentrations of the tested compounds in reaction buffer (20 mM Tris pH 8.5; 150mM NaCl; 0.5 mM TCEP; 5% DMSO and 10% glycerol).

### 4.7. qRT-PCR

Quantitative RT-PCR was employed to quantify the mRNA levels of the AR-V7 splice variant in 22Rv1 cells upon treatment with compounds at 10 and 25 μM concentrations. 22Rv1 cells were starved in RPMI media supplemented with CSS for 48 h, before 24 h treatment with DMSO (0.1%) or compounds. RNA was extracted with TRIzol (Invitrogen, Carlsbad, CA, USA) followed by cDNA synthesis (SuperscriptII, Invitrogen, Carlsbad, CA, USA). RT-PCR (125 ng cDNA, 5 μM primers, Sybr green master mix) was performed on a ViiA 7 thermal cycler. Actin RNA was used as normalized control.

### 4.8. Western Blotting

Western blotting was performed using 22Rv1 cells starved in RMPI with 5% CSS media and treated with compounds for 24 h. The blot was incubated with rabbit anti-actin antibody (1:500 dilution) and mouse anti-AR-NTD 441 monoclonal antibody (1:500 dilution). Visualization of the immune-complexes was done by an enhanced chemiluminescence system (Millipore, Burlington, MA, USA) followed by exposure to X-ray films.

### 4.9. Cell Viability Assay

PrestoBlue assay was used to assess the effect on viability of 22Rv1 cells treated with compounds at increasing concentrations (up to 25 μM) for 3 days. High-sensitivity fluorescence was used as detection method according to the manufacturer’s protocol. PrestoBlue cell viability reagent was purchased from Invitrogen #A-13162 (Invitrogen Molecular Probes, Eugene, OR, USA).
